# Vegetal Compounds as Sources of Prophylactic and Therapeutic Agents in Dentistry

**DOI:** 10.3390/plants10102148

**Published:** 2021-10-10

**Authors:** Raluca-Adriana Milutinovici, Doina Chioran, Roxana Buzatu, Ioana Macasoi, Susan Razvan, Raul Chioibas, Ion Virgil Corlan, Alina Tanase, Calniceanu Horia, Ramona Amina Popovici, Stefania Dinu, Cristina Dehelean, Alexandra Scurtu, Iulia Pinzaru, Codruta Soica

**Affiliations:** 1 Departament of Orthodontics, Faculty of Dental Medicine, Victor Babeș University of Medicine and Pharmacy, 9 Revolutiei 1989 Ave., 300070 Timisoara, Romania; raluca_balan22@yahoo.com; 2 Orthodontic Research Center (ORTHO-CENTER), Faculty of Dental Medicine, Victor Babes University of Medicine and Pharmacy, Revolutiei Ave. 1989 No. 9, 300041 Timisoara, Romania; 3 Department of Dento-Alveolar Surgery, Faculty of Dental Medicine, Victor Babeș University of Medicine and Pharmacy, 9 Revolutiei 1989 Ave., 300070 Timisoara, Romania; chioran.doina@umft.ro; 4 Department of Facial Tooth Aesthetics, Faculty of Dental Medicine, Victor Babeș University of Medicine and Pharmacy, 9 Revolutiei 1989 Ave., 300070 Timisoara, Romania; drbuzaturoxana@gmail.com; 5 Departament of Toxicology and Drug Industry, Faculty of Pharmacy, Victor Babeș University of Medicine and Pharmacy, 2nd Eftimie Murgu Sq., 300041 Timișoara, Romania; cadehelean@umft.ro (C.D.); alexandra.scurtu@umft.ro (A.S.); iuliapinzaru@umft.ro (I.P.); codrutasoica@umft.ro (C.S.); 6 Research Center for Pharmaco-Toxicological Evaluations, Faculty of Pharmacy, “Victor Babes” University of Medicine and Pharmacy, Eftimie Murgu Square No. 2, 300041 Timisoara, Romania; 7 Department of Family Medicine, Faculty of Medicine, Victor Babeș University of Medicine and Pharmacy, 2nd Eftimie Murgu Sq., 300041 Timișoara, Romania; 8 Department of Surgery I, Faculty of Medicine, Victor Babeș University of Medicine and Pharmacy, 2nd Eftimie Murgu Sq., 300041 Timișoara, Romania; office@medcom.ro; 9 Department of Management, Legislation and Communication in Dentistry, Faculty of Dental Medicine, Victor Babeș University of Medicine and Pharmacy, Eftimie Murgu Square No. 2, 300041 Timisoara, Romania; corlan.ionut@yahoo.com (I.V.C.); tanasealinadoina@gmail.com (A.T.); ramona.popovici@umft.ro (R.A.P.); 10 Department of Periodontics, Faculty of Dental Medicine, Victor Babeș University of Medicine and Pharmacy, 9 Revolutiei 1989 Ave., 300070 Timisoara, Romania; horia_calniceanu@yahoo.com; 11 Department of Pedodontics, Faculty of Dental Medicine, Victor Babeș University of Medicine and Pharmacy, 9 Revolutiei 1989 Ave., 300070 Timisoara, Romania; stefania@dr-dinu.com; 12 Departament of Pharmaceutical Chemistry, Faculty of Pharmacy, Victor Babeș University of Medicine and Pharmacy, 2nd Eftimie Murgu Sq., 300041 Timișoara, Romania

**Keywords:** phytocompounds, chemical characterization, biological activity, dentistry

## Abstract

Dental pathology remains a global health problem affecting both children and adults. The most important dental diseases are dental caries and periodontal pathologies. The main cause of oral health problems is overpopulation with pathogenic bacteria and for this reason, conventional therapy can often be ineffective due to bacterial resistance or may have unpleasant side effects. For that reason, studies in the field have focused on finding new therapeutic alternatives. Special attention is paid to the plant kingdom, which offers a wide range of plants and active compounds in various pathologies. This review focused on the most used plants in the dental field, especially on active phytocompounds, both in terms of chemical structure and in terms of mechanism of action. It also approached the in vitro study of active compounds and the main types of cell lines used to elucidate the effect and mechanism of action. Thus, medicinal plants and their compounds represent a promising and interesting alternative to conventional therapy.

## 1. General Aspects

Plant extracts and vegetal compounds used in monotherapy are important sources of therapeutic and prophylactic agents. They are used successfully alone or combined with chemotherapeutic agents. They have specific structures and possess multiple biological and chemical properties [1]. These structures are often used as basic compounds in industrial agent development. Phytochemicals isolated from plants and in rational usage are considered safe and effective. Nowadays, oral products with vegetal compounds included are an important part of modern therapy.

Dental pathology is a global health problem, affecting both underdeveloped and developing or developed countries. The most common oral pathologies are dental caries and periodontal disease, and the most serious oral pathologies are oral and pharyngeal cancer. For these reasons, the World Health Organization considers that oral health is a right of all people [2]. Another issue facing oral health is the large number of children who are affected by tooth decay [3]. There are currently many treatments for oral pathologies, but these have unpleasant side effects, such as altered oral microbiota or systemic gastrointestinal symptoms. For this reason, there is an acute need for new alternatives to conventional treatments, plants being an area of interest. An important example in which plants find their utility is the treatment of bacterial infections at the oral level because most conventional antibiotics are ineffective due to bacterial resistance, and those that are effective have unpleasant side effects [4]. 

Due to this, natural compounds are a safer alternative to antibiotics in the treatment of oral infections and are also an approach in the prevention and treatment of other oral diseases, including dental caries, but also more serious diseases such as cancer.

## 2. Herbal Compounds in Dentistry

Medicinal plants are a topic of interest for current research in the field of medicine, being increasingly used for the treatment of a large number of pathologies. Additionally, many drugs currently used in allopathic medicine have their origin in medicinal plants. So, plants are both sources of traditional medicines and an alternative to them [5].

One of the most basic oral health care processes is brushing your teeth. For this reason, one of the first uses of herbs in dentistry was the obtaining of natural toothbrushes using herbs. Studies show that these “natural brushes” present a large number of therapeutic effects due to the phytochemical composition. Thus, the vitamin C content protects the gums, the tannins provide a deep cleansing of the gingival tissue, and the volatile oils act by stimulating blood circulation.

Another basic practice in dentistry is rinsing the oral cavity. By using herbal mouthwashes, studies have suggested that there is an improvement in oral health, with many phytochemicals having an anti-inflammatory effect, reducing gingival bleeding, but also an antibacterial effect essential for maintaining proper oral health [6].

Dental and oral areas are contaminated generally with germs such as *Streptococcus mutans, Staphylococcus aureus,* or *Candida albicans*. The most common treatments for these aspects are synthetic drugs such as chlorhexidine, triclosan, and fluoride. There are some restrictive problems in toothpaste usage of the fluoridated type for children under six years to avoid changing the pigment of teeth and weakening of enamel. Chlorhexidine as a chlorophenyl bisbiguanide causes also pigmentation of the oral environment. They induce an altered sense of taste and oral dryness and irritation as well as negative systemic aspects and scaling of gingival. For triclosan, it seems it does not bind well on the oral site because of its strong positive charge. Another challenge in modern formulations included their potential to destroy carcinogenic microorganisms. Series of vegetal compounds and extracts developed such an important activity. The classical antibiotics could determine microbial resistance for this category [7].

Besides the microbial contamination of the oral cavity, another important aspect in dental pathology is represented by viral infections. Viral infections have many implications for oral health, primarily due to the pathologies they can cause, but also due to contamination that can be bidirectional. Thus, the microorganism can be transferred from the patient to the medical team, but also in reverse. The main types of viruses involved that are found in the oral cavity and that can cause alterations in health are herpes viruses and liver viruses B, C, and D [8]. Regarding the classic antiviral treatment, it is often inefficient, with many viruses not responding to conventional treatment, and vaccination is currently limited to a small number of microorganisms. Due to these drawbacks, and in addition, due to the fact that conventional drugs are quite expensive and viruses have frequent mutations making them resistant to treatment, compounds of natural origin may be a suitable alternative for the treatment of viral infections [9].

**Figure 1:**
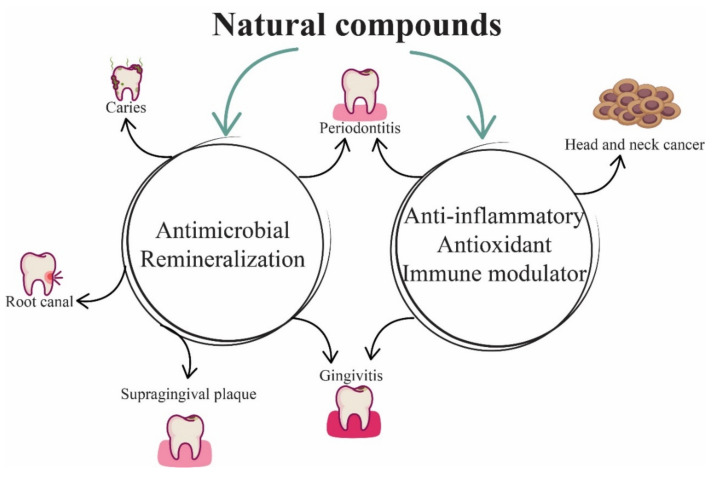
The main biological actions and therapeutic uses of natural compounds in dentistry.

For these reasons, medicinal plants are increasingly used in dentistry due to the ingredients they contain. The plants used in dental pathology have different therapeutic actions such as anti-inflammatory, antimicrobial, antifungal, antiviral, and analgesic. Based on these multiple therapeutic actions, natural compounds find their utility in a multitude of dental pathologies (Figure 1).

## 3. Vegetal Sources of Compounds on Dentistry Products

Although progress is being made in the field of dentistry in the treatment of oral diseases, dental pathology is still a public health problem. The most common pathologies encountered in the dental field are dental caries and periodontal diseases [10]. However, oral health is also linked to a number of systemic pathologies, such as diabetes [11].

Medicinal plants have been used for thousands of years in traditional medicine, and today, in underdeveloped countries, phytotherapy remains the main method of treatment [12]. Although recent studies have focused on the discovery of new natural compounds for the treatment of various pathologies, only 1% of plants with therapeutic potential are known, which leaves room for future research. One of the areas in which natural compounds find their utility is the treatment of bacterial infections, given that modern medicine is currently facing a crisis of increasing resistance to conventional antibiotics [13]. In terms of composition, plants have over 2000 compounds that can be classified according to their chemical structure into four major groups: terpenoids, nitrogen compounds, sulfur compounds, and phenolic compounds [14,15].

Regarding the role of terpenoids in dentistry, they are used mainly due to the antibacterial activity on the bacteria responsible for the formation of tooth decay [7]. Thus, diterpenes, a subgroup of terpenoids, are known for their strong antibacterial activity on Staphylococcus aureus [16]. In addition to this antibacterial activity, studies have suggested that diterpenes may act as a potentiator of the antibacterial activity of conventional antibiotics [17]. In addition, studies that have focused on bacteria associated with tooth decay have suggested that diterpenes such as ent-kauran [18] and ent-pimarane [19] have strong antibacterial activity on Streptococcus mutans, Streptococcus salivarius, Streptococcus mitis, and Lactobacillus casei species. The mechanism underlying the antibacterial action is not fully elucidated, but studies have suggested that it is based on the ability of diterpenes to cause bacterial lysis and cause cell membrane damage [20]. Thus, terpenoids are a group of substances of interest for the discovery of new sources of anticaries agents. Certain representatives of the terpenoids class, such as monoterpenoids or borneol, are of medical interest due to their antiviral effects. Isoborneol is a terpenoid compound found in a variety of volatile oils, having a strong antiviral effect on herpes simplex virus-1. It has been observed that in vitro, isoborneol causes virus inactivation after approximately 30 min of exposure, without affecting viral adsorption [21]. Additionally, other natural compounds found in essential oils, such as thymol, citral, and γ-terpinene have been tested for their antiviral effect, noting that it is similar to the effect of acyclovir, a classic antiviral used in infection with herpes simplex virus-1. The advantage of natural compounds is that they are also active on viral strains that have become resistant to acyclovir, which suggests that phytocompounds have a different mechanism of action than conventional medicine. The possible mechanism of antiviral action associated with monoterpenoids may be the direct inactivation of the virus and damage to the structures necessary for the adsorption of the virus to the host cell [22].

Phenolic compounds are a complex class of phytocompounds, which have many beneficial therapeutic activities in dentistry such as dentin remineralization, antibacterial, anti-inflammatory, and antioxidant [23]. An example of phenolic compounds useful in dentin remineralization and used clinically for this purpose are proanthocyanidins. They act by interacting with collagen fibers, thus causing the formation of a stable interaction between resin and dentin [24]. Additionally, another phenolic compound, quercetin, increases the resistance of the interaction between resin and dentin, and, in addition, it has useful antioxidant effects in the therapy of dental pathologies [25]. Regarding the antibacterial activity of polyphenolic compounds, it has been tested and confirmed in various studies. Phenolic compounds act on the main bacterial species responsible for the appearance of dental pathologies such as: Streptococcus mutans [26] and Aggregatibacter actinomycetemcomitans [27]. The mechanism of antibacterial action is based on the ability of phenolic compounds to inhibit the activity of the enzyme glucosyltransferase [26]. Additionally, antibacterial activity is correlated with the ability of phenolic compounds to alter the redox balance in the bacterial cell [28]. Phenolic compounds find their utility in a variety of dental pathologies due to the numerous therapeutic actions they possess. In the class of polyphenolic compounds, the category of flavonoids is of real interest. Regarding their antiviral activity, scientific documentation dates back to the early 1990s, when studies showed that the combination of acyclovir and apigenin causes increased antiviral activity on the herpes simplex viruse types 1 and 2 [29]. Concerning the mechanism of action of flavonoids, this is a complex one, with the involvement of multiple biological targets involved in the production of viral infection. If we turn our attention to hepatitis B and C viruses, studies show that flavonoids have the ability to inhibit viral replication by affecting enzymes involved in this process, such as protease NS3 and polymerase NS5B [30].

High-proline proteins represent over half of the total protein contained in salivary excretion. In addition to the beneficial effects of the polyphenolic compounds discussed above, it is worth paying special attention to analyzing the interaction between them, especially tannins, with food proteins, because these interactions can have harmful effects on the health of the oral cavity. Thus, polyphenols can cause the formation of covalent complexes with globular proteins thus leading to the formation of complexes, the stabilization of proteins and their precipitation [31]. These reactions between polyphenols and high-proline protein are harmful to oral health because this category of protein found in the salivary composition has the role of calcium binding, inhibiting the formation of hydroxyapatite and mediating the binding of hydroxyapatite, thus being part of the dental-acquired films. On the other hand, the interaction between polyphelonols and high-proline protein can be beneficial because these proteins are also involved in the binding of hydroxyapatite bacteria, thus contributing to the formation of dental floss [32].

Alkaloids are another class of phytocompounds used in dentistry due to their therapeutic actions. Thus, alkaloids such as berberine show antibacterial activity on *A. actinomycetemcomitans* and *P. gingivalis* species, and it is observed that the mechanism of action consist in inhibition of collagenase enzyme activity [33]. Additionally, alkaloids find their practical utility in dentistry as local anesthetics, being used in various dental interventions [34]. These compounds also have a beneficial effect in viral infections due to their ability to stimulate the host’s immune system by increasing interferon synthesis and stimulating macrophage activity. Research in the field has shown that various plant extracts with high alkaloid content have shown intense antiviral activity on a multitude of viral species such as hepatitis A, B, C, and D and herpes simplex virus [35].

In conclusion, plants are an important source for compounds useful in dentistry and have the potential to be used as therapeutic agents or prevention of dental disease (Table 1).

## 4. Herbs as Therapeutic Agents in Dentistry

**Figure 2:**
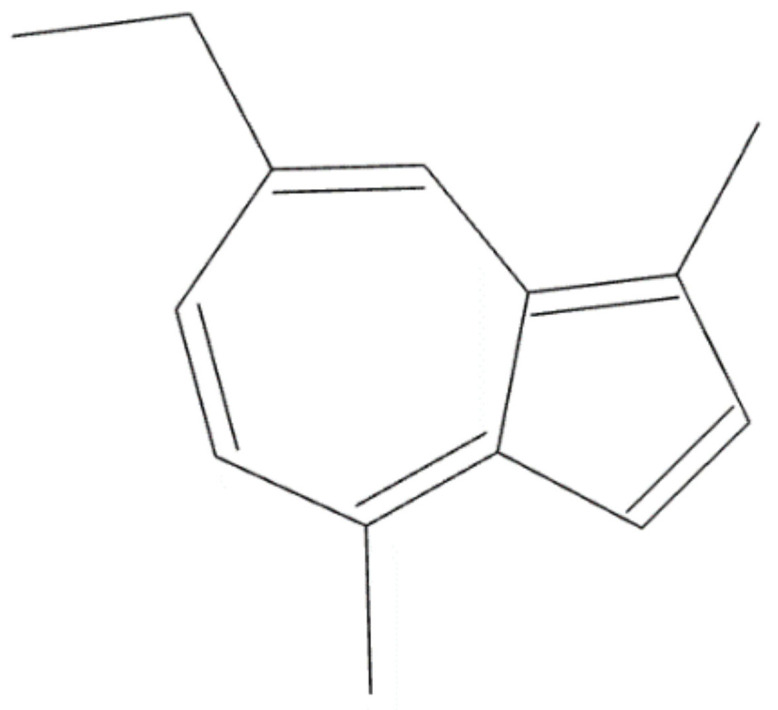
Chemical structure of chamazulene.

*Achillea millefolium* L. belongs to the Asteraceae family and has been used in traditional medicine for centuries [50], having many beneficial therapeutic properties, including anti-inflammatory, anti-ulcer and anti-cancer effects [51]. Considering the phytochemical composition, *Achillea millefolium* is rich in flavonoids, alkaloids, terpenes, tannins, phenolic acids, etc., the most important constituents being represented by flavonoids and phenolic acids. The main constituent found in essential oil from *A. millefolium* oil is chamazulene (Figure 2) [52]. Regarding the beneficial effects of *A. millefolium* in dental pathology, it has been observed that the extract has beneficial effects in healing of oral mucositis, a major complication of classical antitumor chemotherapy [53]. Responsible for this therapeutic effect are considered to be the flavonoids and tannins contained in *A. millefolium* [54].

**Figure 3:**
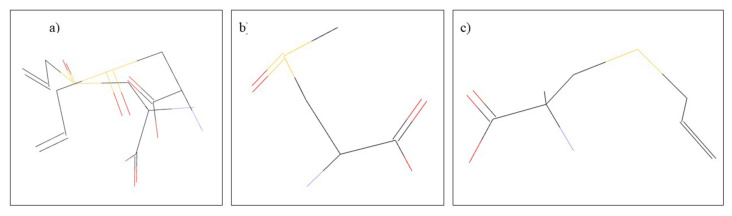
Chemical structure of: (**a**) allin, (**b**) methiin, and (**c**) S-allylcysteine.

*Allium sativum* L., or garlic, belongs to the genus Allium, the Alliaceae family, and possesses many well-known therapeutic properties, which are due to the main constituents represented mainly by alliin, methiin, and S-allylcysteine (Figure 3) [55]. Garlic is known for its beneficial effects in common pathologies such as colds or hypertension [56], but also for its beneficial immunostimulatory properties in the treatment of cancer and heart and lung diseases [55]. In dentistry, garlic finds its utility due to its antibacterial effects in the treatment of periodontitis, dental caries, endodotitis, and recent studies have also shown the beneficial effect of garlic in the treatment of oral cancer [57]. The antibacterial effects of *A. sativum* have been studied both in vitro and in vivo. Thus, in a study of the multi-drug-resistant species of *S. mutans*, it was observed that the garlic has an inhibition effect on bacterial growth [58]. Another therapeutic effect of garlic beneficial in dental pathology is represented by its antifungal effect. Thus, the antifungal effect of a garlic extract was compared with that of nystatin and fluconazole, noting that garlic extract has a more pronounced antifungal effect than that of conventional antifungal drugs [59,60]. Regarding the antitumor effects, they were studied in vitro, and it was observed that S-allylcysteine has an antiproliferative effect on human oral squamous cancer cells [61]. Underlying the bacteriostatic mechanism of action is the conversion of allicin to diallyldisulfide in the presence of the allinelyase enzyme, which causes inhibition of bacterial DNA and RNA synthesis [62].

*Aloe Vera* L. is a plant that belongs to the Liliaceae family and contains a variety of phytoconstituents such as vitamins A, C, E, and group B, amino acids, enzymes, and sugars. As for *Aloe vera* gel, it consists of over 70 constituents, the most important being lignin. In addition, *Aloe vera* contains various chemical compounds responsible for the anti-inflammatory effects of the plant, such as bradykinase and salicylic acid [63]. The main dental pathologies in which Aloe vera finds its utility include: oral lichen planus [64], stomatitis [65], oral submucosal fibrosis [66], oral mucositis [67], gingivitis [68], periodontitis [69], and alveolar osteitis [70]. The beneficial effects in oral pathology are based on the following biological activities of *Aloe vera*: (i) the bactericidal effect on the main cariogenic and periodontopathic bacteria [71]; (ii) antimicrobial effect on resistant species found in the pulp space, such as *Candida albicans* or *Eterococcus faecalis*, having the advantage that Aloe vera-based extracts can be used intracanal due to sedative and lubricating effects [72]; (iii) antioxidant properties beneficial in the treatment of oral submucous fibrosis [66] and (iv) immunostimulatory effects by stimulating macrophages [67].

*Calendula officinalis* L. is known by the popular name of “pot marigold” and is part of the Asteraceae family. It is an intensely used plant in traditional medicine due to its numerous therapeutic properties such as: anti-inflammatory, re-epithelializing, antibacterial, antifungal, antioxidant, and immunomodulatory [73]. The main phytocompounds found in *Calendula officinalis* are flavonoids, tannins, polysaccharides, phenolic acids, tannins and saponosides [74]. The main dental pathologies on which the effect of *Calendula officinalis* was studied and on which an improvement was observed were plaque and gingivitis. In the randomized study by Khairan et al. [74], they showed that tincture of *Calendula officinalis* causes a decrease in plaque and gingivitis [74]. In addition, Iauk et al. [75] have shown that *Calendula officinalis* flower extract has antibacterial effects on the most important species involved in dental pathogenesis, and Zilda et al. [76], showed that calendula has nystatin-like antifungal effects, and its use in oral candidiasis is useful. Due to the content of polysaccharides and mucilages of the calendula, it has a good bio-adhesion in porcine mouth membranes, which suggests even if it could be used in the treatment of canker sores, foot ulcers or gingivitis [77]. Another beneficial effect of calendula worth mentioning is the reduction of oropharyngeal mucositis in patients undergoing radiotherapy [78]. This therapeutic property is due to the increase in hyaluronic acid deposits due to the flavonoids contained in calendula [79]. The main biological actions that give utility to calendula in dental treatment can be summarized as follows: (i) anti-inflammatory action by reducing proinflammatory cytokines [80]; (ii) antioxidant effects due to the content of flavonoids and carotenoids [81] and (iii) immunomodulatory effects due to the content of polysaccharides [76].

**Figure 4:**
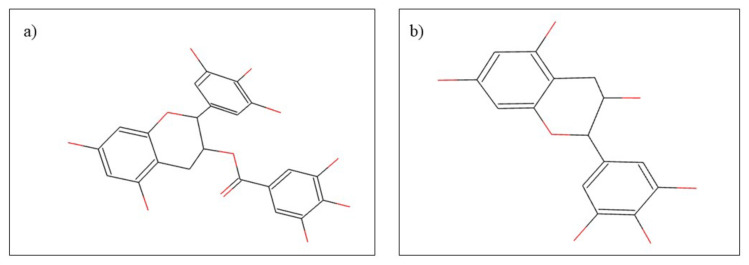
Chemical structure of (**a**) epigallocatechin-3-gallate and (**b**) epigallocatechin.

*Camellia sinensis* L. is also known as “green tea” and is part of the Theaceae family. Green tea is intensely consumed globally, reaching the order of billions of cups consumed daily. From a phytochemical point of view, *Camellia sinensis* is distinguished by its rich content in proteins, vitamins, and minerals, but especially by the content of catechin. The most important catechins contained by green tea are: epigallocatechin-3-gallate and epigallocatechin. The content rich in phytocompounds offers green tea many therapeutic properties useful both in dental pathologies and in other pathologies such as cardiovascular illnesses or cancer [82]. From a dental point of view, green tea has multiple therapeutic effects beneficial in diseases such as gingivitis, periodontitis, dental caries, halitosis, and even oral cancer [83]. Gingivitis and periodontitis are two pathologies characterized by the appearance of bacterial overpopulations in the gingiva and tooth margins. In the case of these pathologies, it was highlighted that the regular consumption of green tea through its catechins content causes a decrease in the development of bacterial species such as *Porphyromonas gingivalis* or *Prevotella nigrescens* thus promoting healing [82,84]. Regarding the effect of green tea in the prevention of dental caries, it has two major mechanisms of action. First of all, due to its fluoride content, *Camellia sinensis* contributes to the remineralization of dental tissue and inhibits the growth of pathogenic bacteria [85]. The second mechanism of action is based on the content of epigallocatechin 3-gallate, epicatechin and epicatechin gallate (Figure 4). In a study by Otake et al. [86], they demonstrated that the polyphenols contained in green tea prevent the adhesion of *Streptococcus mutans* to the tooth surface [86]. Epigallocatechin 3-gallate also has a beneficial role in the treatment of halitosis. The deodorizing action is based on the reduction of hydrogen sulfide and methyl mercaptan, the main compounds responsible for the appearance of halitosis [87].

**Figure 5:**
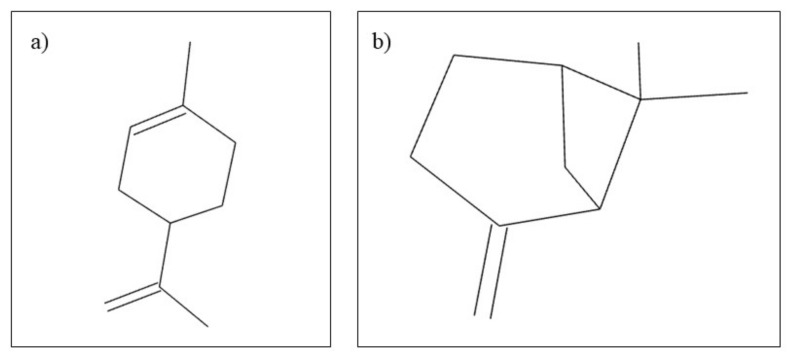
Chemical structure of (**a**) D-Limonene and (**b**) B-pinene.

*Citrus aurantifolia,* also known as lime, belongs to the Rutaceae family. In terms of its use, lime has both beneficial medical and cosmetic effects. Regarding the chemical composition, it can be stated that *Citrus aurantifolia* is rich in carbohydrates, alkaloids, flavonoids, steroids, and anthraquinones [88]. In general medicine and dentistry, volatile lime oil is used. Volatile oils are known for their therapeutic actions such as antimicrobial, antifungal, antitumor, and anti-inflammatory [13]. Volatile lime oil contains mainly D-Limonene and B-pinene (Figure 5) [89]. The effects of volatile lime oil have been tested in dentistry especially for antimicrobial effects. Thus, in the study by Aripin et al. [89], they compared the antimicrobial effects on *Streproccocus mutans* species of several types of Citrus spp. It was observed that volatile lime oil has the highest antibacterial activity compared to volatile oils from *C. limon, C. hystrix, C. sinensis* and *C. nobilis*. This antibacterial activity is beneficial for the treatment of dental caries [89]. In a similar study, the antibacterial effect of aqueous and alcoholic lime extracts on the main bacterial species causing caries was studied, noting that *Citrus aurantifolia* has antibacterial effects on *C. pneumonia, S. aureus* and *P. mirabilis* species [88]. These results are supported by similar studies [90] in which similar antibacterial effects of lime extract were observed, and, in addition, Pathan et al. [91], demonstrated that *C. aurantifolia* has antimicrobial effects on *E. coli* species.

*Cocos nucifera* L. is known as the coconut and is part of the Arecaceae family. The most important product obtained from coconut is coconut oil, known for its therapeutic effects which are antibacterial, antifungal and antiviral. Coconut water is also a product obtained from coconut and intensively used in dentistry due to its rich content in natural sugars and minerals [92]. From a compositional point of view, coconut water contains over 90% water. Besides water, coconut water contains micronutrients, vitamins, amino acids, and enzymes, but the most important components are phytohormones (cytokinins and auxin). The most important compound from the auxin class found in coconut water is indole-3-acetic acid [93]. Due to this composition, coconut water is used in dentistry, generally as a tissue growth agent and as a suitable cellular medium for maintaining the viability of cells and the periodontal ligament. In addition, due to the fact that it is sterile, coconut water can be used as a means of transport in case of emergency for a sprained tooth [94]. Additionally, coconut oil is rich in fatty acids such as lauric, myristic, caprylic, and palmitic acid. In dentistry, coconut oil is used for rinsing the mouth, which is favored due to its viscosity which aids the elimination of food, bacteria, and microorganisms from the oral cavity [95].

**Figure 6:**
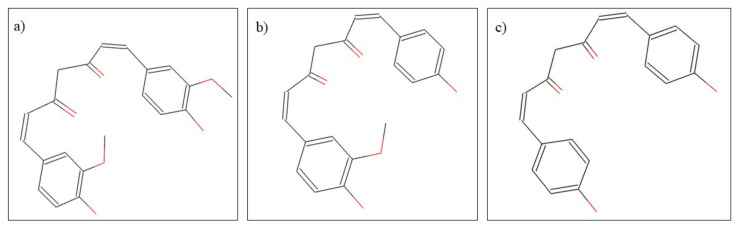
Chemical structure of (**a**) diferuloylmethane, (**b**) demethoxycurcumin, and (**c**) bisdemethoxycurcumin.

*Curcuma longa* L. is popularly known as turmeric and belongs to the Zingiberaceae family. Regarding the chemical composition, turmeric contains a multitude of phytocompounds, the most important for its therapeutic actions being diferuloylmethane, demethoxycurcumin, and bisdemethoxycurcumin (Figure 6). Turmeric has uses both in the food industry for its effects of improving storage conditions and time, and in medicine, especially due to its anti-inflammatory, analgesic, antitumor, carminative, antiseptic, and antibacterial properties [96]. In dentistry, it is used in both systemic and local pathologies such as gingivitis, periodontitis [97], dental caries [98], head and neck cancer [99], as a subgingival irrigant [100], oral submucous fibrosis [101], and oral lichen planus [102]. The use of turmeric in dentistry is based on its many therapeutic actions. For the treatment of gingivitis and periodontitis, turmeric treatment is based on its anti-inflammatory effect. Chatterjee et al. [103], conducted a double-blind study in which it was observed that the anti-inflammatory effects of turmeric can be used to improve the action of chlorhexidine [103]. Additionally, in dentistry, turmeric is used due to its antibacterial properties against *Streptococcus mutans*. In a study by Lee et al. [98], it was observed that turmeric has a dose-dependent antibacterial action and at high doses also has the ability to inhibit the formation of *Streptococcus mutans* biofilm and thus prevent the formation of dental caries [98]. Additionally, due to the antibacterial activity of turmeric, it can be used as an intracanal drug, preventing the formation of pathogens such as Enterococcus faecalis [104]. Regarding the efficacy of turmeric in the treatment of head and neck cancer, and of oral submucous fibrosis, pilot studies were performed, in vivo, in which it was observed that turmeric has positive effects and can be used as a therapeutic agent in these pathologies [99,101].

**Figure 7:**
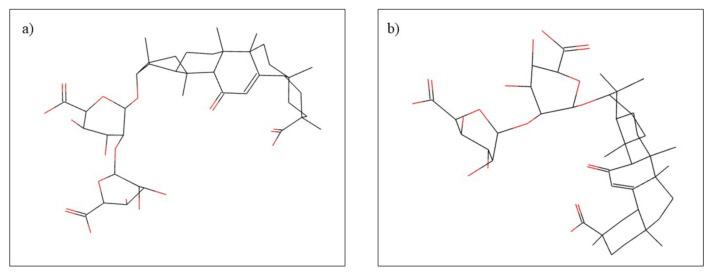
Chemical structure of (**a**) glycyrrhizic acid and (**b**) glycyrrhizin.

*Glycyrrhiza glabra* L. is part of the Leguminosae family and has been intensely used in traditional medicine and Ayurvedic medicine for over 4000 years [105]. The most used part of the plant in medicine is the root. It contains a multitude of phytocompounds such as liquirtin, isoliquertin liquiritigenin and rhamnoliquirilin, flavonoids (prenyllicoflavone A), and saponins (glycyrrhizic acid, glycyrrhizin) (Figure 7) [106]. In traditional medicine, *G. glabra* has many therapeutic effects, from antiviral, anti-tumor, anti-diabetic to antidepressant and antiepileptic [107], and in dentistry, it is used in the treatment of dental caries, gingival and periodontal diseases, oral candidiasis, oral cancer, and as an endodontic treatment [108]. Due to its glabridine content, *G. glabra* has strong antibacterial effects on both Gram-positive and-negative bacterial species. In addition, other constituents such as glycrrhizin and glycyrrhizol-A have been shown to be beneficial in preventing caries [109]. In addition, the phytocompounds Licochalcone A and 18 alpha-glycyrrhetinic acid have antibacterial effects on the species *Porphyromonas gingivalis* preventing the formation of biofilm and stimulating the host immune system, thus having beneficial effects in the treatment of gingivitis and periodontitis [110]. In addition to the antibacterial effect, *G. glabra* extract has also proven effective in the treatment of oral candidiasis by inhibiting the formation and growth of biofilm due to the content of licochalcone A, glabridin, and liquiritigenin which have antifungal effects on *C. albicans* [106].

Grape seed extract. Grapes are one of the most widely grown plants in the world. Recently, grape seeds have been increasingly used in medicine due to their rich content of phenolic compounds and strong antioxidant properties, effective in treating many diseases such as ulcers, diabetes, hypercholesterolemia, bacterial infections, and tumors [111]. In dentistry, grape seeds are mainly used for restorative dental work and prevention of dental caries. The main compound of grape seeds responsible for the beneficial actions in dental pathologies is represented by the proanthocyanidin group [112]. In vivo studies have shown that grape seed extract has the ability to reduce colonization of *S. mutans*, the main bacterium involved in tooth decay [113]. Additionally, there are numerous studies in the literature that have highlighted the beneficial role of grape seeds in dental reminalization. The mechanisms of action underlying this therapeutic property consist of: (i) grape seed extract mediates remineralization; (ii) grape seed extract prevents dental degradation and (iii) grape seed extract influences the dental tensile strength. These mechanisms are due to the ability of phytocompounds in grape extract to bind to enzymes responsible for tooth enamel degradation and to promote crosslinking of collagen-rich dentin surfaces [112].

*Hypericum perforatum* L. belongs to the Hypericaceae family and contains a multitude of phytocompounds such as flavonoids, tannins, volatile oils, and naphthodianthrones responsible for its therapeutic actions such as anti-inflammatory, antidepressant, antidiabetic, and wound healing [114]. In dentistry, the main action of *Hypericum perforatum* extract is antibacterial. In a study conducted to evaluate the antibacterial effect on the main bacterial species responsible for the production of dental pathologies, it was observed that the aqueous extract has a strong antibacterial effect on all bacterial species [115]. In another study comparing the antibacterial effect of *Hypericum perforatum* volatile oil with that of two solutions of povidone-iodine and chlorhexidine, it was observed that on the bacterial species *Aggregatibacter actinomycetemcomitans*, the essential oil has an antibacterial action similar to that of chlorhexidine solution, and on the species *Porphyromonas gingivalis,* the antibacterial activity was lower than that of the two solutions. However, by combining the essential oil with povidone-iodine and chlorhexidine solutions, a preparation with increased antibacterial activity is obtained, which suggests that *Hypericum perforatum* essential oil causes an increase in the antibacterial activity of the solutions [116].

**Figure 8:**
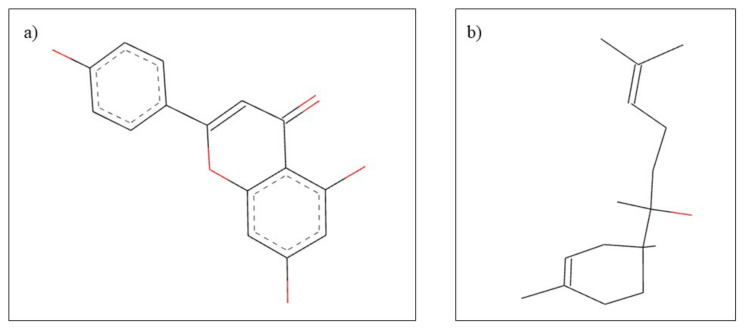
Chemical structure of (**a**) apigenin and (**b**) α-bisabolol.

*Matricaria chamomilla* L. from the Asteraceae family, has been used in traditional medicine for thousands of years, being one of the most important medicinal plants in Europe [117] with various therapeutic effects including anti-inflammatory, antioxidant, antibacterial and relaxant properties [118]. Numerous phytocompounds have been identified in *Matricaria chamomilla L*., the main classes are represented by volatile terpenoids, sesquiterpenes, coumarins, flavonoids, and phenolic acids [119]. The most important constituents for therapeutic use in dentistry are apigenin, α-bisabolol (Figure 8), chamazulene, and umbelliferone. Regarding the therapeutic effects in the field of dental pathologies, it has been reported that chamomile extract has beneficial properties in oral mucositis and gingivitis, effects due to phenolic compounds, especially apigenin [120,121,122].

**Figure 9:**
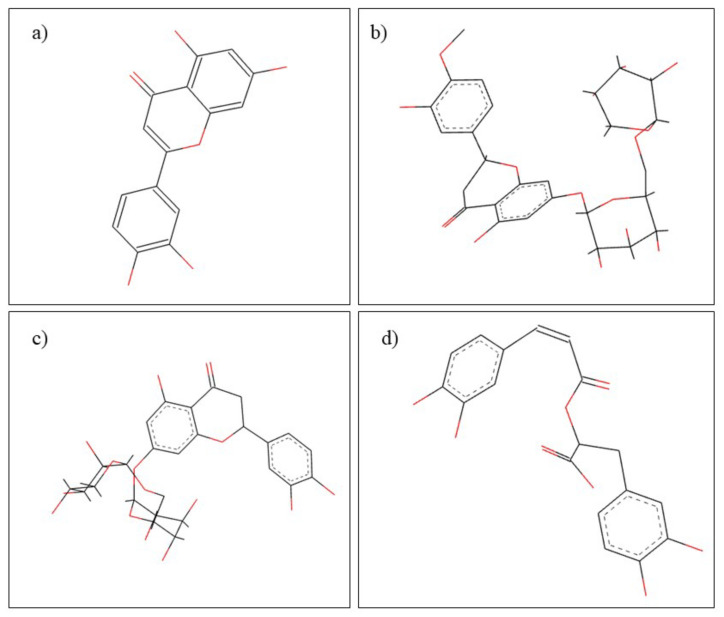
Chemical structure of (**a**) luteolin, (**b**) hesperidin, (**c**) eriocitrin, and (**d**) rosmarinic acid.

*Mentha piperita* L. is a medicinal plant that belongs to the Lamiaceae family, and is known for its uses in traditional medicine and for its distinctive aroma [123] and having different biological properties, including anti-inflammatory, antioxidant, antimicrobial, antiviral, and antitumor effects [124]. Regarding the phytochemical composition, peppermint contains flavonoids, phenolic acids, volatile compounds, lignans, and stilbenes, the most abundant compounds being luteolin, hesperidin, eriocitrin, and rosmarinic acid (Figure 9) [125]. In folk medicine, *M. piperita* oil has been used to reduce gingival inflammation and to stop toothache [120]. Raghavan et al. [126] conducted a study in which it was demonstrated the antimicrobial action of *M. piperita* on oral microorganisms such as *Streptococcus mutans*, *Candida albicans,* and *Aggregatibacter actinomycetemcomitans* that can cause serious oral disease. Following this study, we can consider *Mentha piperita* as an alternative to the conventional treatment of infections, with its antibiotic resistance being more and more common [126].

**Figure 10:**
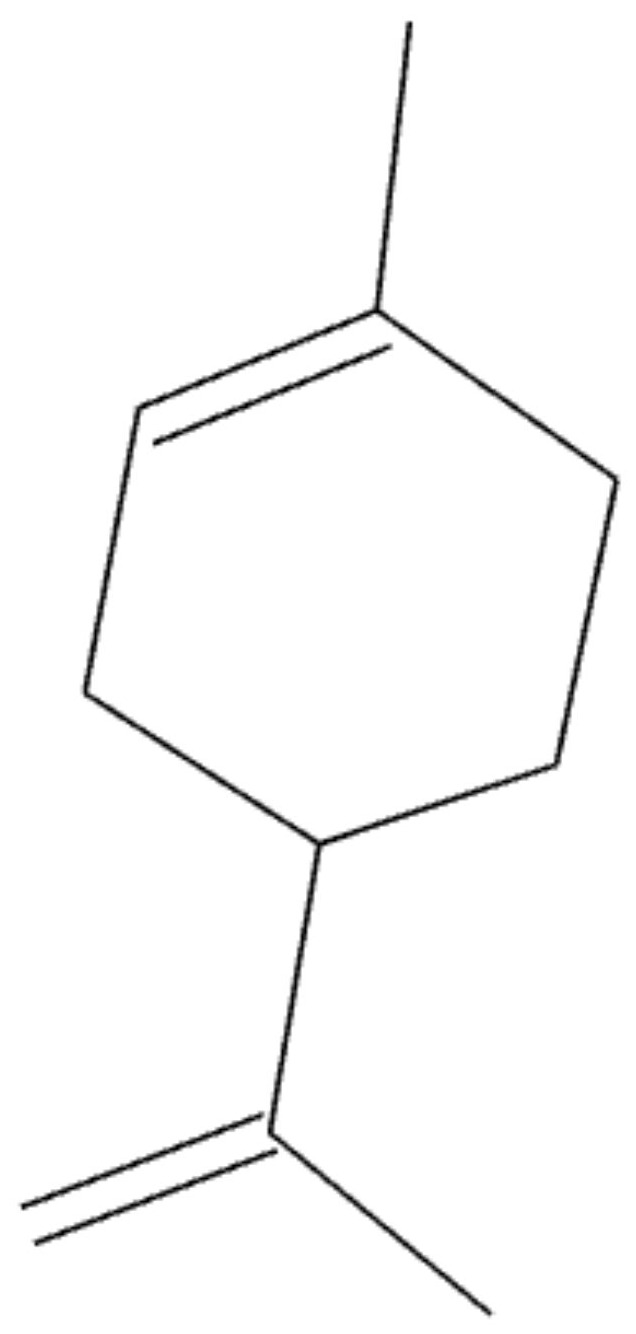
Chemical structure of D-limonen.

Orange oil is a natural product extracted from the peel of *Citrus sinensis* from the Rutaceae family. The composition of the oil is vast, comprising hydrocarbons, esters, aldehydes, and alcohols, the most important phytocompound being D-limonene (Figure 10) [127]. Following various studies, it has been observed that orange oil has multiple benefits, including antioxidant, antimicrobial, and anti-aflatoxigenic properties [128,129]. In the field of dentistry, in a study conducted by Yadav et al. [130], it was observed that both orange and eucalyptus oil can be an alternative to xylene in dissolving different endodontic sealers [130].

Papain is a proteolytic enzyme obtained from the latex of the papaya fruit (*Carica papaya*) which belongs to the Caricaceae family [131]. The enzyme has been used for a long time for its analgesic and anti-inflammatory properties in the treatment of injuries, traumas, and allergies. In addition, its activity in dentistry regarding the removal of dental caries has been reported, by its action of breaking down partially degraded collagen from infected tissues [132,133].

**Figure 11:**
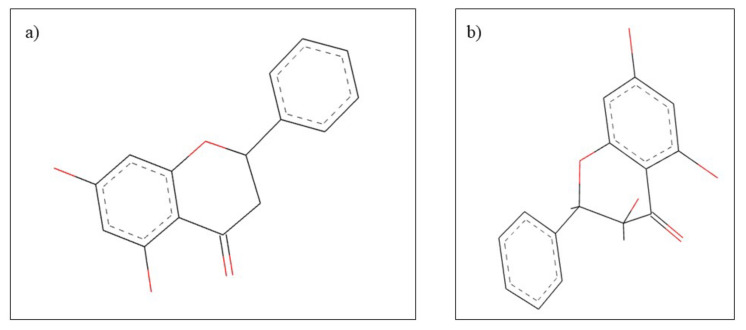
Chemical structure of (**a**) pinocembrin and (**b**) pinobanksin.

Propolis is a resinous material extracted from various plants by bees [134]. In terms of chemical composition, propolis is a substance rich in resins, essential oils, wax, vitamins, minerals but also in organic compounds such as flavonoids: pinocembrin, pinobanksin (Figure 11), phenolic compounds, terpenes [135,136]. According to numerous research, propolis has various benefits including antioxidant, wound-healing, antimicrobial, anti-inflammatory, and immunomodulatory effects [137]. In dental practice, propolis has many uses: the treatment of stomatitis, periodontitis, and halitosis. It is also used in dental caries, traumatic ulcers, dentinal hypersensitivity, candidal infections and tooth coating preparations [138,139,140].

**Figure 12:**
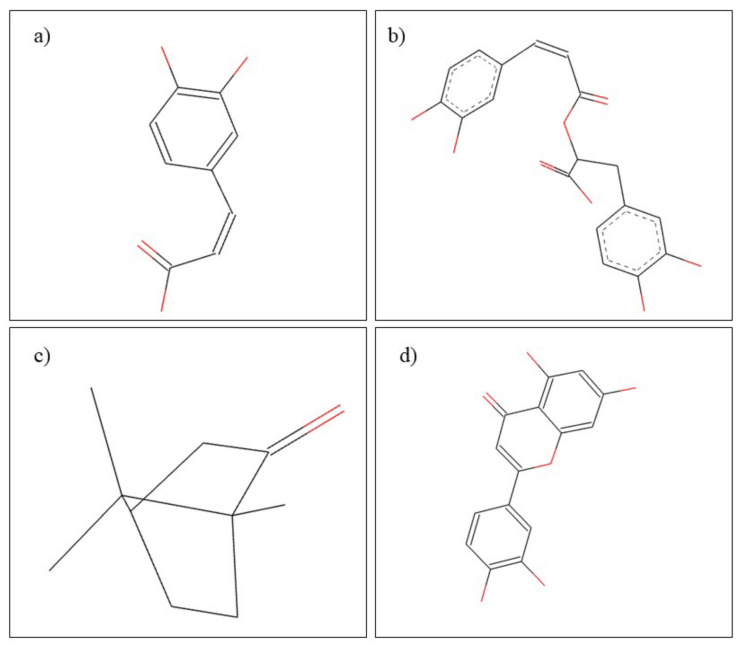
Chemical structure of (**a**) caffeic acid, (**b**) rosmarinic acid, (**c**) camphor, and (**d**) luteolin.

*Rosmarinus officinalis* L. is a widespread plant from the Lamiaceae family, with uses in the treatment of diseases and in the culinary arts [141]. Hundreds of phytocompounds have been identified in the leaves and oil extracted from rosemary, among which the most important are: caffeic acid, rosmarinic acid, camphor, 1,8-cineole, and luteolin (Figure 12) [142]. Regarding the therapeutic properties of the species *Rosmarinus officinalis* L., it has been proven to be used particularly for its carminative, antispasmodic, analgesic at the muscular level, and expectorant action [143,144]. In dental pathology, *Rosmarinus officinalis* L. is used to treat gingival diseases and to refresh the mouth [120].

Septilin is a complex polyherbal preparation from Himalaya Drug Company that contains six medicinal plants and minerals, with indications for treating acute and chronic infections, even those of a dental nature [145,146], due to the antibacterial, anti-inflammatory, and immunomodulatory activity of the phytocomplex [147].

**Figure 13:**
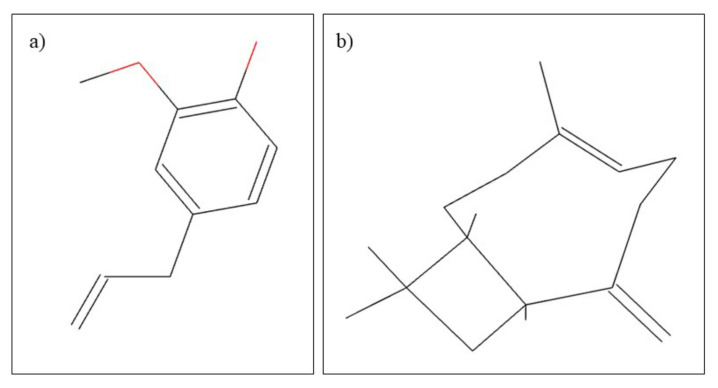
Chemical structure of (**a**) eugenol and (**b**) β-caryophyllene.

*Syzygium aromaticum* L. is a medicinal plant that belongs to the Myrtaceae family with uses in the food industry as a spice but also with important uses in curative medicine [148]. Various phytoconstituents have been identified in the extracts of *Syzygium aromaticum* L., the main representatives being phenolic molecules, monoterpenes, and sesquiterpenes; while clove oil is rich in eugenol and β-caryophyllene (Figure 13) [149,150]. Many studies have been reported on the therapeutic benefits of *Syzygium aromaticum*, the antioxidant activity being significant due to the high content of phenolic compounds. In addition, the species has strong antimicrobial and antiviral activity as well as insecticidal and antitumor action, especially observed in the oil extracted from leaf buds [150,151,152,153]. In dentistry, the volatile oil is used to refresh the mouth, to reduce dental pain but also to treat gingival bleeding, while gel preparations are used for anesthetic action [154].

**Figure 14:**
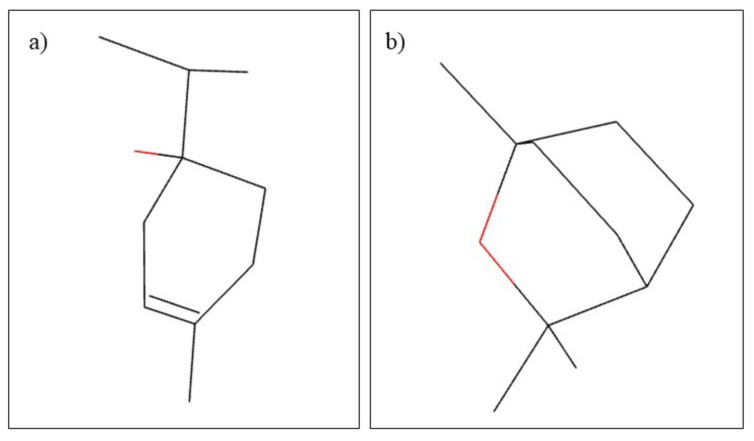
Chemical structure of (**a**) terpinen-4-ol and (**b**) 1,8-cineole.

Tea tree oil is a volatile oil, obtained from the leaves of *Melaleuca alternifolia*, a plant from Australia that belongs to the Myrtaceae family, known for uses in alternative and complementary medicine [155]. Considering the phytochemical composition, tea tree essential oil comprises over 100 active compounds, including terpinen-4-ol and 1,8-cineole (Figure 14), with many therapeutic effects, such as antibacterial, antiviral, antifungal, antiprotozoal activities, wound healing and anticancer effects [156]. Regarding the benefits in dentistry, it has been shown that the volatile oil used in mouthwash preparations has anti-inflammatory action on the gingival tissues but also oral antiseptic effect [154,157]. In addition, it has a possible effect in treating the root canal due to its solvent properties, useful in dissolving the necrotic pulp [120].

**Figure 15:**
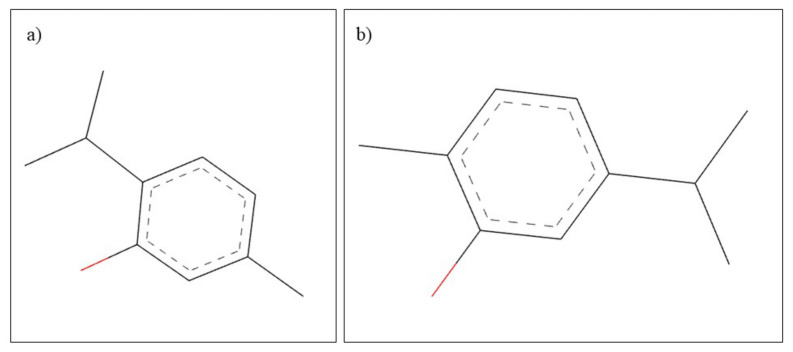
Chemical structure of (**a**) thymol and (**b**) carvacrol.

*Thymus vulgaris* L., from the Lamiaceae family, has been known since ancient times for its culinary and pharmacological properties [158]. The main therapeutic benefits consist of antiseptic, antispasmodic, carminative, diuretic, and expectorant actions, and the effects are due to the vast composition of the species in polyphenolic compounds, especially thymol and carvacrol (Figure 15) [159,160,161]. Regarding the effectiveness of *Thymus vulgaris* L. extracts in dental diseases, the antimicrobial action of volatile oil has been demonstrated, even on the species *Streptococcus mutans*, one of the microorganisms responsible for dental caries. The extract of thyme can also be used for the treatment of oral herpes, along with other herbs [162]. These beneficial therapeutic properties occur due to the content of thymol and carvacrol [163].

**Figure 16:**
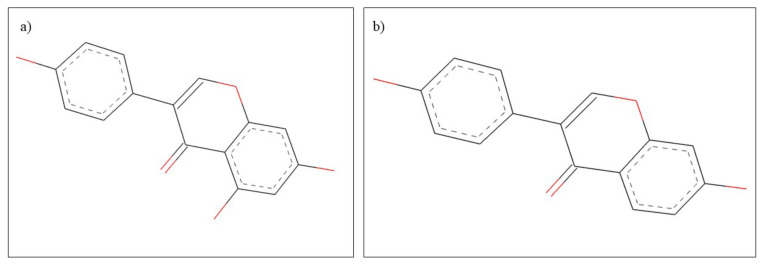
Chemical structure of (**a**) genistein and (**b**) daidzein.

*Trifolium pratense* L., from the Fabaceae family, is a well-known plant, cultivated for fodder purposes as a nitrogen fixer, which improves the soil. Besides its usage in agriculture, the plant has been used in traditional medicine in various diseases: it relieves osteoporosis, reduces cholesterol, and treats the symptoms of menopause. Responsible for these effects are the phytoestrogens and isoflavones genistein, and daidzein (Figure 16), contained in *Trifolium pratense* L. [164]. In the dental field, it has been observed that *Trifolium pratense* extracts act as an antibiotic and anti-inflammatory in inflamed gums, and inflammation due to either an abscess or a dental procedure [6].

## 5. In Vitro Experimental Studies

**Figure 17:**
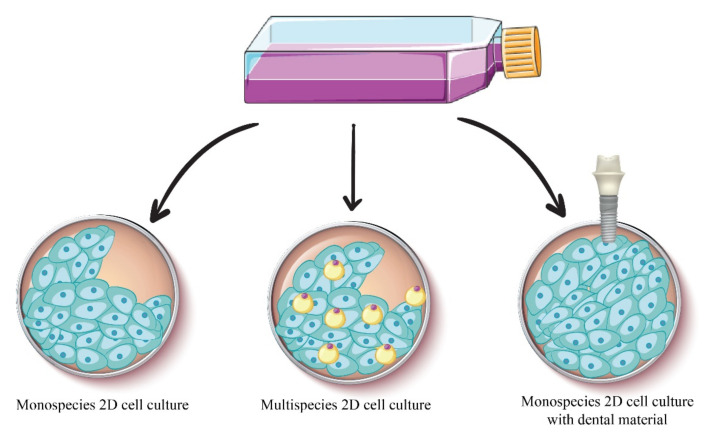
The main types of cell cultures used in dentistry.

In vitro experimental studies are an important part of the development of new therapies used in medicine and dentistry, having the advantage that they provide a number of essential information for further in vivo and clinical studies [165]. In vitro studies are used both in the dental field and in other fields, such as cancer [166]. In vitro studies show the ability to simulate the oral environment and provide standard and controllable conditions for conducting experiments. The design of in vitro studies varies from simple to complex, depending on the purpose of the research (Figure 17) [167]. The main pathologies that can be reproduced in vitro to study the effect of the compounds on them include dental caries [168], gingivitis [169], and periodontal diseases [170].

Given that the oral environment is characterized by the presence of many saprophytic bacteria and most dental pathologies are based on overpopulation with bacterial cultures, in vitro methods offer the advantage of allowing direct study of the interaction between bacteria and cells, providing an accurate picture of the cellular response [171]. For example, cell cultures can be used to study the inflammatory process and response of different eukaryotic cells in the presence of pathogenic bacterial species [172]. Such a study by Yee et. al. [173] allows the evaluation of the response of eukaryotic cells to the presence of *P. gingivalis* species, one of the bacteria involved in the appearance and development of periodontitis. In a similar study, Yilmaz et. al., evaluated the response of primary gingival epithelial cells in the presence of *P. gingivalis*, noting that in the presence of pathogenic bacteria cell adhesion is stimulated by targeting specific cellular pathways [174]. Another bacterial species with implications in the development of periodontitis is *A. actinomycetemcomitans*. In an in vitro study, *A. actinomycetemcomitans* was applied to a cancer cell line using different environmental conditions, so it was observed that cell adhesion is influenced by both the host environment and the culture conditions [175]. Such studies are needed to more closely observe how pathogens interact with cellular targets, thus providing useful information for the development of new therapies.

Another approach to in vitro studies is the use of a co-cultures system, useful for comparing the effects of different pathogens on cells at the same time [171]. Thus, an in vitro study on human gingival epithelial cells explored the effect exerted by six Gram-negative bacterial strains responsible for the development of periodontal diseases. The study allowed the comparison of the aggressiveness of bacterial strains and the observation of their interaction with specific cellular targets [176].

In addition, cell co-cultures can be used in dentistry, in which several types of eukaryotic cells are grown together. One such study is the one conducted by Bodet et al. [177], in which epithelial cells were cultured together with macrophages to observe in more detail how they respond in the presence of the bacterial species *P. gingivalis*. Another more recent study used three cell types, dentritic cells, gingival epithelial keratinocytes, and T-cells, which were analyzed for their response to the bacterial species *P. gingivalis* [178]. In such studies, an important factor to consider is the ratio of cell types that are co-culture.

Cell cultures are also useful for evaluating the biocompatibility of a dental material, such as implants. Thus, human fibroblasts can be cultured directly on the dental materials together with the bacterial species of interest [179]. Additionally, the technique has been developed to allow the study of biocompatibility directly in a 96-well plate, using in parallel bacterial species to evaluate its mode of action [180].

**Figure 18:**
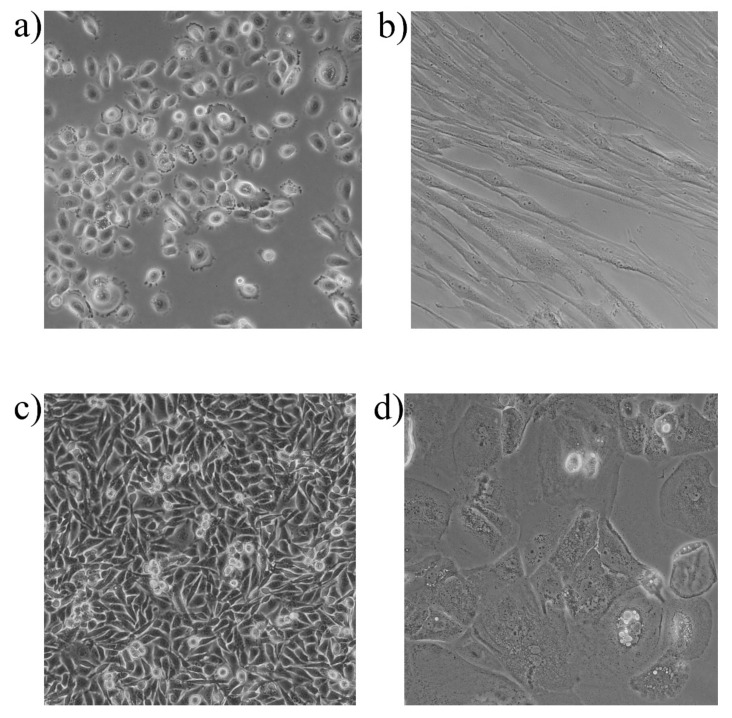
Morphological aspect of some of the most frequently used cell lines in the dental field: (**a**) primary gingival keratinocytes (PGK); (**b**) human primary gingival fibroblasts (HGF); (**c**) human keratinocyte (HaCaT) and (**d**) human tongue squamous cell carcinoma (SCC4). 20× magnification.

One factor worth considering when conducting such studies is the origin of eukaryotic cells. In the dental field, several eukaryotic cells can be used, such as primary human gingival epithelial cells, fibroblasts, immortalized human gingival, skin keratinocyte cell lines, but also oral carcinoma cell lines (Figure 18) [171]. Considering the numerous studies performed on cell cultures, it can be concluded that this technique is useful and easy to perform, allowing the evaluation of specific interactions that occur in various dental pathologies, as well as the evaluation of potential new therapies.

## 6. Conclusions

The plant kingdom remains an unexplored field in terms of the use of plants in the treatment of human pathologies. Due to the need to find new therapeutic alternatives in the dental field, plants and compounds of natural origin represent a real interest due to their high efficacy and low toxicity. One of the most important biological activities presented by phytocompounds is the antibacterial action, useful in the treatment of most oral health problems. An advantage of medicinal plants is that they have a complex antibacterial mechanism of action, which is why the resistance of bacteria is diminished. An important field of research in the phytotherapeutic field is represented by in vitro studies that allow the creation of an environment similar to the biological one, thus allowing the study of the effects and mechanisms of action of phytocompounds. Thus, medicinal plants are still a field worth studying due to the many beneficial properties on human health and due to the low side effects compared to conventional therapy.

It is essential that medicinal plants be private from a scientific and objective point of view. The use of medicinal plants is still a common practice in human medicine. The advantage of using natural extracts stems from the synergistic effect of compounds existing in plants that potentiate their activity causing an improved therapeutic effect. At the same time, phytocompounds can cause serious adverse reactions that deserve special attention from both physicians and patients. It is of real importance that in the therapy, to be used, the origin of medicinal plants must be known exactly, and in the case of compounds isolated from plants, they must be carried out in an appropriate quantitative and qualitative control.

In the future, research directions should be directed to developed countries, where the rate of bacterial and viral resistance is increased, and dental damage is a major health problem. Natural compounds should also be analyzed in combination with other phytocompounds, as well as with conventional drugs currently used in therapy to observe in more detail the synergistic effect between them.

## Figures and Tables

**Table 1:** Examples of natural compounds and their use in dentistry.

Natural Compounds	Dentistry Applications	References
α-thujone, and β-thujone	Antimicrobial activity against *S. mutans*, *L. rhamnosus* and *A. viscosus* Anticandidal activities Antiplaque activity	[36]
Allicin	Antimicrobial activity against *S. mutans*, *S.sobrinus*, and *A. oris*	[37]
Carvone	Treatment of gingivitis and periodontal disease	[5]
Catechins	Antibacterial activity against *S. mutans* and *S. sobrinus*	[38]
Chitosan	Antihemorrhagic Analgesic Anti-inflamatory activity Antibacterial and antifungal activity	[39]
Cinnamonic acid	Periodontitis treatment Decreases periodontal inflammation Antibacterial activity against *S. mutans* and *L. casei*	[40,41]
Curcumin	Antioxidant and anti-inflamatory activity Analgesic in dental pain	[42]
Eugenol	Antibacterial activity against *P. gingivalis* Antibiofilm activity against *C. albicans* and *S. mutans* Anti-inflammatory activity	[41,43]
Gingerol	Anti-inflamatory activity Antioxidant activity Antimicrobial activity against *P. gingivalis* and *P. endodontalis* Treatment of recurrent apthous stomatitis, xerostomia, dental caries and gingivitis	[44]
Linalool	Antibacterial activity against *P. gingivalis* and *S. mutans*	[41]
Menthol	Antiplaque and anti-gingivitis agent	[45]
Sanguinarine	Gingivitis and periodontal disease	[46]
tt-Farnesol	Antimicrobial activity against *S. mutans*	[47]
Rosmarinic acid	Antimicrobial activity on Gram-negative and positive bacteria (*S. aureus*, *S. albus*, *V. cholerae* and *E. coli*)	[5]
Thymol	Antibacterial activity against *S. mutans*	[48]
Quercetin	Treatment of cancers, periodontal disease, oral lesions, tooth decay, and oral infections	[49]
